# A matched-pair analysis comparing 5x4 Gy and 10x3 Gy for metastatic spinal cord compression (MSCC) in patients with favorable survival prognoses

**DOI:** 10.1186/s13014-015-0403-y

**Published:** 2015-04-15

**Authors:** Dirk Rades, Stefan Huttenlocher, Theo Veninga, Amira Bajrovic, Michael Bremer, Volker Rudat, Steven E Schild

**Affiliations:** Department of Radiation Oncology, University of Lübeck, Ratzeburger Allee 160, 23538 Lübeck, Germany; Department of Radiation Oncology, Dr. Bernard Verbeeten Institute, Tilburg, The Netherlands; Department of Radiation Oncology, University Medical Center Eppendorf, Hamburg, Germany; Department of Radiation Oncology, Hannover Medical School, Hannover, Germany; Department of Radiation Oncology, Saad Specialist Hospital, Al Khobar, Saudi Arabia; Department of Radiation Oncology, Mayo Clinic, Scottsdale, AZ USA

**Keywords:** Metastatic spinal cord compression, Irradiation, Favorable survival prognoses, Fractionation schedules, Treatment outcomes

## Abstract

**Background:**

It is currently not possible to get an approval of our ethics committee for a randomized trial cmparing 5x4 Gy and 10x3 Gy for MSCC that includes patients with favorable survival prognoses. Therefore, this matched-pair study following strict matching criteria was perfomed instead.

**Methods:**

In this study, 142 receiving 5x4 Gy were retrospectively matched (1:1) to 142 patients receiving 10x3 Gy with respect to ten characteristics. These characteristics included age, gender, performance status, tumor type, involved vertebrae, other bone metastases, visceral metastases, interval between tumor diagnosis and MSCC, pre-RT ambulatory status, and time developing motor deficits.

**Results:**

On multivariate analysis, post-RT motor function was associated with performance status (p < 0.001), tumor type (p < 0.001), and time developing motor deficits (p < 0.001). RT was successful in 76% of patients receiving 5x4 Gy and 69% receiving 10x3 Gy (p = 0.14). Pre.RT ambulatory status showed a strong trend with respect to local control (LC) of MSCC in the multivariate analysis (p = 0.058). 1-year LC rates were 87% after 5x4 Gy and 93% after 10x3 Gy (p = 0.16). On multivariate analysis, survival (OS) was associated with performance score (p < 0.001), visceral metastases (p < 0.001), and pre-RT ambulatory status (p = 0.004). 1-year OS rates were 68% after 5x4 Gy and 73% after 10x3 Gy (p = 0.64).

**Conclusions:**

In patients irradiated for MSCC who had favorable survival prognoses, post-RT motor function, LC and OS were not significantly different after 5x4 Gy and after 10x3 Gy.

## Background

Five to 10% of adult cancer patients experience metastatic spinal cord compression (MSCC) during the course of their malignant disease, which is considered an oncologic emergency [1,2]. The vast majority of these patients receive radiotherapy (RT), either alone or preceded by decompressive surgery. Carefully selected patients can benefit from the addition of surgery, whereas most patients worldwide presenting with MSCC are treated with RT alone [3]. Such a palliative situation like MSCC requires an individualized treatment approach, including the most appropriate fractionation schedule of RT. A variety of fractionation schedules are in use for MSCC ranging from single-fraction RT to fractionated schedules given over weeks. For example, RT with a short overall treatment time would be ideal for patients with a poor estimated survival time, if it was as effective as more time consuming schedules.

Until now, only three randomized trials exist that compared different fractionation schedules for MSCC. Two studies compared two fractions of 8 Gy (with one week rest between the two fractions) to either a split-course regimen (3x5 Gy followed by one week rest and 5x3 Gy given over one week) or 1x8 Gy [4,5]. In both of these trials, the compared fractionation schedules resulted in similar treatment outcomes. The most frequently used fractionation schedules for MSCC are 5x4 Gy over one week and 10x3 Gy over two weeks. Therefore, it appears reasonable to compare these schedules in a randomized trial. Such a trial was begun and preliminary results presented last year at the annual ESTRO meeting [6]. Due to ethical considerations this trial was limited to patients with a poor or intermediate survival prognosis, since retrospective studies suggested that patients with more favorable survival prognoses benefit from longer-course RT such as 10x3 Gy in terms of better local control of MSCC. However, such retrospective studies always bear the risk of selection bias. Patients considered to have a more favorable survival prognosis may have received preferentially 10x3 Gy rather than 5x4 Gy. Nevertheless, the results of the retrospective studies shouldn’t be ignored. Therefore, it is currently not possible to get an approval of our ethics committee for a randomized trial comparing 5x4 Gy and 10x3 Gy for MSCC that includes patients with more favorable survival prognoses. Because further studies defining the optimal fractionation schedule of RT for MSCC in patients with a more favorable prognosis are required, we performed a matched-pair study with strict matching criteria in an attempt to minimize the inclusion of biases.

## Patients and methods

In this matched-pair study, two fractionation schedules were compared in patients with MSCC and more favorable survival prognoses. The patients had a score of more than 35 points in a validated survival score [7,8]. That score considered six prognostic factors found to be significantly associated with OS in a multivariate analysis. These factors included the type of primary tumor, other bone metastases at the time of RT, visceral metastases at the time of RT, interval between tumor diagnosis and MSCC, ambulatory status prior to RT, and time developing motor deficits prior to RT. The score for each factor was obtained from the 6-month survival rates divided by 10. The survival score for each patient represented the sum of the six factor scores and ranged from 20 to 45 points. In the simplified version of the scoring system, three prognostic groups were defined: 20–30 points (poor survival prognosis), 31–35 points (intermediate survival prognosis), and 36–45 points (more favorable survival prognosis).

The present study was approved by the local ethic committee (University of Lübeck). We retrospectively matched (1:1) 142 patients receiving 5x4 Gy in one week to 142 patients receiving 10x3 Gy in two weeks with respect to ten patient characteristics. The ten characteristics included age (≤63 versus ≥ 64 years, median age: 63.5 years), gender, Eastern Cooperative Oncology Group (ECOG) performance status (1–2 versus 3–4), type of primary tumor (breast cancer versus prostate cancer versus myeloma/lymphoma versus lung cancer versus other tumors), number of involved vertebrae (1–2 versus ≥ 3), other bone metastases at the time of RT (no versus yes), visceral metastases at the time of RT (no versus yes), interval between tumor diagnosis and MSCC (≤15 months versus >15 months), ambulatory status prior to RT (not ambulatory versus ambulatory), and time developing motor deficits prior to RT (1–7 versus 8–14 versus >14 days). The distributions of the patient characteristics are summarized in Table 1.

**Table 1:** **Distribution of the potential prognostic factors in the two treatment groups**

	**5 x 4 Gy**	**10 x 3 Gy**
	**N patients (%)**	**N patients (%)**
**Age**		
≤63 years (N = 142)	71 (50)	71 (50)
≥64 years (N = 142)	71 (50)	71 (50)
**Gender**		
Female (N = 138)	69 (49)	69 (49)
Male (N = 146)	73 (51)	73 (51)
**ECOG performance status**		
1-2 (N = 226)	113 (80)	113 (80)
3-4 (N = 58)	29 (20)	29 (20)
**Type of primary tumor**		
Breast cancer (N = 110)	55 (39)	55 (39)
Prostate cancer (N = 76)	38 (27)	38 (27)
Myeloma/lymphoma (N = 40)	20 (14)	20 (14)
Lung cancer (N = 12)	6 (4)	6 (4)
Other tumors (N = 46)	23 (16)	23 (16)
**Involved vertebrae (N)**		
1-2 (N = 134)	67 (47)	67 (47)
≥3 (N = 150)	75 (53)	75 (53)
**Other bone metastases at the time of RT**		
No (N = 140)	70 (49)	70 (49)
Yes (N = 144)	72 (51)	72 (51)
**Visceral metastases at the time of RT**		
No (N = 264)	132 (93)	132 (93)
Yes (N = 20)	10 (7)	10 (7)
**Interval from tumor diagnosis to MESCC**		
≤15 months (N = 80)	40 (28)	40 (28)
>15 months (N = 204)	102 (72)	102 (72)
**Ambulatory status before RT**		
Not ambulatory (N = 30)	15 (11)	15 (11)
Ambulatory (N = 254)	127 (89)	127 (89)
**Time developing motor deficits before RT**		
1-7 days (N = 20)	10 (7)	10 (7)
8-14 days (N = 68)	34 (24)	34 (24)
>14 days (N = 196)	98 (69)	98 (69)

Further criteria for inclusion were MSCC of the thoracic or lumbar spine, no prior surgery or RT to the spinal regions involved by MSCC, and confirmation of MSCC by spinal computed tomography or spinal magnetic resonance imaging. RT was performed with linear accelerators to the spinal segments involved by MSCC plus one normal vertebra above and below the metastatic lesions. If the spinal lesions extended to the cervical spine, two normal vertebrae above the metastatic lesions were included.

The grading of each patient’s motor function was performed with a 5-point scale [9]: Grade 0: normal strength; Grade 1: ambulatory without aid, Grade 2: ambulatory with aid, Grade 3: not ambulatory, Grade 4: paraplegia. The ambulatory status has been routinely assessed in the centers participating in this study. Improvement or deterioration of motor function was defined as a change of at least one point. The fractionation schedule and the ten additional patient characteristics were analyzed with respect to the success of RT. Successful RT was defined either as improvement of motor function by at least one point or as maintaining the status “ambulatory without aid”. The investigated 11 factors were evaluated in a multivariate manner with a logistic regression analysis.

LC was defined as freedom from an in-field recurrence of MSCC in the irradiated spinal segments. An in-field recurrence had to be confirmed by spinal imaging. LC and OS rates were counted from the last day of RT. For the univariate analyses of LC and OS, the Kaplan-Meier-method [10] and the log-rank test were used. The characteristics identified as significant (p < 0.05) on univariate analysis were subsequently analyzed in a multivariate manner with the Cox proportional hazards model. If applicable, two separate analyses were performed including either ambulatory status or ECOG performance status, since both are confounding variables. The patients were followed until death or for median 14 months (range: 6 to 96 months) in those alive at the last follow-up.

## Results

According to the multivariate analysis, the effect of RT on motor function was significantly associated with the ECOG performance status (p < 0.001), the type of primary tumor (p < 0.001), and the time developing motor deficits prior to RT (p < 0.001). The success of RT with respect to motor function was not significantly associated with the fractionation schedule of RT (p = 0.14). RT was successful in 76% of patients receiving 5x4 Gy and in 69% of patients receiving 10x3 Gy, respectively. The results of the analysis of the RT effect on motor function are summarized in Table 2.

**Table 2:** **Impact of potential prognostic factors on motor function**

	**Radiotherapy successful**	**Radiotherapy not successful**	**P**
	**N patients (%)**	**N patients (%)**	
**RT regimen**			
5 x 4 Gy (N = 142)	108 (76)	34 (24)	
10 x 3 Gy (N = 142)	98 (69)	44 (31)	0.14
**Age**			
≤63 years (N = 142)	104 (73)	38 (27)	
≥64 years (N = 142)	102 (72)	40 (28)	0.77
**Gender**			
Female (N = 138)	110 (80)	28 (20)	
Male (N = 146)	96 (66)	50 (34)	0.51
**ECOG performance status**			
1-2 (N = 226)	177 (78)	49 (22)	
3-4 (N = 58)	29 (50)	29 (50)	*<0.001*
**Type of primary tumor**			
Breast cancer (N = 110)	93 (85)	17 (15)	
Prostate cancer (N = 76)	51 (67)	25 (33)	
Myeloma/lymphoma (N = 40)	29 (73)	11 (27)	
Lung cancer (N = 12)	10 (83)	2 (17)	
Other tumors (N = 46)	23 (50)	23 (50)	*<0.001*
**Involved vertebrae (N)**			
1-2 (N = 134)	95 (71)	39 (29)	
≥3 (N = 150)	111 (74)	39 (26)	0.49
**Other bone metastases at the time of RT**			
No (N = 140)	101 (72)	39 (28)	
Yes (N = 144)	105 (73)	39 (27)	0.28
**Visceral metastases at the time of RT**			
No (N = 264)	193 (73)	71 (27)	
Yes (N = 20)	13 (65)	7 (35)	0.68
**Interval from tumor diagnosis to MESCC**			
≤15 months (N = 80)	50 (63)	30 (37)	
>15 months (N = 204)	156 (76)	48 (24)	0.12
**Ambulatory status before RT**			
Not ambulatory (N = 30)	18 (60)	12 (40)	
Ambulatory (N = 254)	188 (74)	66 (26)	0.47
**Time developing motor deficits before RT**			
1-7 days (N = 20)	7 (35)	13 (65)	
8-14 days (N = 68)	43 (63)	25 (37)	
>14 days (N = 196)	156 (80)	40 (20)	*<0.001*

The p-values were obtained from the multivariate analysis.

Pre-RT ambulatory status was significantly associated with LC of MSCC in the univariate analysis (p = 0.027) and showed a strong trend in the multivariate analysis (p = 0.058). The time of developing motor deficits prior to RT was significant in the univariate analysis of LC (p = 0.028) but not in the multivariate analysis (p = 0.60). The fractionation schedule had no significant association with LC (p = 0.16). The 1-year LC rates were 87% after 5x4 Gy and 93% after 10x3 Gy, respectively. The results of the univariate analysis of LC of MSCC are shown in Table 3, and the results of the multivariate analysis in Table 4.

**Table 3:** **Impact of potential prognostic factors on local control of MSCC (univariate analysis)**

	**At 6 months**	**At 12 months**	**P**
**RT regimen**			
5 x 4 Gy (N = 142)	95	87	
10 x 3 Gy (N = 142)	98	93	0.16
**Age**			
≤63 years (N = 142)	97	92	
≥64 years (N = 142)	97	87	0.84
**Gender**			
Female (N = 138)	98	93	
Male (N = 146)	95	86	0.27
**ECOG performance status**			
1-2 (N = 226)	97	91	
3-4 (N = 58)	96	82	0.11
**Type of primary tumor**			
Breast cancer (N = 110)	99	93	
Prostate cancer (N = 76)	95	84	
Myeloma/lymphoma (N = 40)	100	95	
Lung cancer (N = 12)	100	100	
Other tumors (N = 46)	91	84	0.16
**Involved vertebrae (N)**			
1-2 (N = 134)	98	93	
≥3 (N = 150)	96	87	0.09
**Other bone metastases at the time of RT**			
No (N = 140)	96	91	
Yes (N = 144)	97	88	0.53
**Visceral metastases at the time of RT**			
No (N = 264)	97	90	
Yes (N = 20)	100	80	0.32
**Interval from tumor diagnosis to MESCC**			
≤15 months (N = 80)	97	97	
>15 months (N = 204)	97	88	0.24
**Ambulatory status before RT**			
Not ambulatory (N = 30)	96	71	
Ambulatory (N = 254)	97	91	*0.027*
**Time developing motor deficits before RT**			
1-7 days (N = 20)	78	78	
8-14 days (N = 68)	98	96	
>14 days (N = 196)	98	89	*0.028*

**Table 4:** **Multivariate analysis of local control of MSCC**

	**Risk ratio**	**95%-confidence interval**	**P-value**
**Ambulatory status before radiotherapy**	2.88	0.96 – 7.04	0.058
**Time of developing motor deficits before radiotherapy**	1.19	0.60 – 2.12	0.60

In the univariate analysis of survival, OS was associated with ECOG performance status (p < 0.001), visceral metastases at the time of RT (p < 0.001), and ambulatory status prior to RT (p = 0.006). The fractionation schedule had no significant impact on OS (p = 0.64). The 1-year OS rates were 68% after 5x4 Gy and 73% after 10x3 Gy, respectively. The results of the univariate analysis of OS are summarized in Table 5. In the multivariate analysis, OS was significantly associated with ECOG performance score (p < 0.001), visceral metastases at the time of RT (p < 0.001), and ambulatory status prior to RT (p = 0.004). Results of the multivariate analysis of OS are summarized in Table 6.

**Table 5:** **Impact of potential prognostic factors on overall survival (univariate analysis)**

	**At 6 months**	**At 12 months**	**P**
**RT regimen**			
5 x 4 Gy (N = 142)	87	68	
10 x 3 Gy (N = 142)	84	73	0.64
**Age**			
≤63 years (N = 142)	90	73	
≥64 years (N = 142)	81	68	0.29
**Gender**			
Female (N = 138)	87	74	
Male (N = 146)	84	67	0.20
**ECOG performance status**			
1-2 (N = 226)	92	77	
3-4 (N = 58)	60	43	*<0.001*
**Type of primary tumor**			
Breast cancer (N = 110)	89	80	
Prostate cancer (N = 76)	80	71	
Myeloma/lymphoma (N = 40)	78	63	
Lung cancer (N = 12)	83	56	
Other tumors (N = 46)	93	50	0.11
**Involved vertebrae (N)**			
1-2 (N = 134)	89	69	
≥3 (N = 150)	83	71	0.42
**Other bone metastases at the time of RT**			
No (N = 140)	91	72	
Yes (N = 144)	80	68	0.10
**Visceral metastases at the time of RT**			
No (N = 264)	88	73	
Yes (N = 20)	50	35	*<0.001*
**Interval from tumor diagnosis to MESCC**			
≤15 months (N = 80)	80	63	
>15 months (N = 204)	88	73	0.25
**Ambulatory status before RT**			
Not ambulatory (N = 30)	63	50	
Ambulatory (N = 254)	88	73	*0.006*
**Time developing motor deficits before RT**			
1-7 days (N = 20)	85	73	
8-14 days (N = 68)	79	59	
>14 days (N = 196)	88	74	0.23

**Table 6:** **Multivariate analysis of overall survival**

	**Risk ratio**	**95%-confidence interval**	**P-value**
**ECOG performance status**	2.76	1.69 – 4.39	*<0.001*
**Visceral metastases at the time of radiotherapy**	4.47	2.44 – 7.70	*<0.001*
**Ambulatory status before radiotherapy**	2.49	1.36 – 4.26	*0.004*

Acute toxicity such as nausea, diarrhea and radiation dermatitis did not exceed grade 1 according to CTCAE 4.0 in both groups. Late radiation toxicity, particularly radiation myelopathy, was not observed.

## Discussion

MSCC is a palliative situation requiring an individual treatment approach tailored to each patient’s personal situation and needs. The vast majority of these patients do receive RT. One important question is whether the patient would likely benefit from the addition of decompressive surgery plus stabilization of the involved vertebrae prior to RT? Ten years ago, a small randomized trial including 101 patients demonstrated a benefit for carefully selected patients who had a favorable performance status, a survival prognosis of at least a few months, involvement of only one spinal segment by MSCC, and a solid tumor not considered very radiosensitive [3]. The effect on the patients’ ambulatory status was better after the combined approach than after RT alone with post-treatment ambulatory rates of 84% and 57%, respectively (p = 0.001). The median OS times were 4.2 months and 3.3 months, respectively (p = 0.033). Taking into account the specific selection criteria used for this trial, only 10-15% of all patients presenting with MSCC would be suitable candidates for upfront decompressive surgery. Therefore, most patients with MSCC receive RT alone. Although high-precision techniques such as radiosurgery, SBRT and IMRT become more popular for the treatment of painful vertebral metastases [11-13], these techniques are generally not recommended fpr MSCC outside clinical trials [14,15]. This means that conventional RT is the most common modality used for treating MSCC.

If conventional RT is administered, the fractionation schedule should be carefully selected to meet the patient’s individual situation and prognosis. Fractionation schedules applied for MSCC include single-fraction, short-course multi-fraction and longer-course programs. Several retrospective and prospective studies have shown that fractionation schedule has no significant impact on the effect of RT on post-treatment motor function and ambulatory status. Therefore, at least those patients who are estimated to have a short remaining life span appear good candidates for receiving either single-fraction RT or short-course multi-fraction RT. For better prognosis patients who are likely to live six months or longer following RT, longer-course programs appear more appropriate because two large retrospective studies and a prospective study (SCORE-1) did suggest a better LC of MSCC for longer-course RT with 10x3 Gy, 15x2.5 Gy or 20x2 Gy when compared to 1x8 Gy or 5x4 Gy [16-18]. In the SCORE-1 study, the rates of LC of MSCC at one year were 81% and 61%, respectively (p = 0.005) [18]. However, the SCORE-1 study was not randomized. Furthermore, 1x8 Gy and 5x4 Gy were combined to the short-course RT group, and 10x3 Gy, 15x2.5 Gy and 20x2 Gy were combined to the longer-course RT group. The two most frequently used fractionation schedules for MSCC, 5x4 Gy and 10x3 Gy, have been directly compared for the first time in a prospective randomized trial named SCORE-2 [6]. Preliminary results of that trial were presented at the annual ESTRO meeting last year. According to these preliminary results, 5x4 Gy and 10x3 Gy were not significantly different with respect to effect on motor function, post-RT ambulatory status, pain relief, LC of MSCC and OS. However, the SCORE-2 trial is limited to patients with a poor or intermediate survival prognosis based on a validated survival score. The risk of developing an in-field recurrence of MSCC increases with the patient’s remaining lifetime. Thus, patients with favorable survival prognoses are at a higher risk of developing a recurrence than those patients with a poor or intermediate survival prognosis. Because previous studies suggested worse LC after short-course than after longer-course RT, patients with favorable survival prognoses were not included in the SCORE-2 trial [16-18]. Since a direct comparison of 5x4 Gy and 10x3 Gy is an important issue for all patients with MSCC, we performed the present matched-pair study following strict matching criteria in order to further contribute to answering the question whether 10x3 Gy is really superior to 5x4 Gy, or if both schedules are similarly effective in patients with a favorable survival prognosis?

According to the results of the present study, 5x4 Gy and 10x3 Gy were not significantly different with respect to the effect on post-RT motor function, LC of MSCC and OS. In other words, 5x4 Gy appeared not inferior to 10x3 Gy in patients with MSCC and favorable survival prognoses. The results of the present study support performing an additional randomized trial comparing 5x4 Gy and 10x3 Gy that is not strictly limited to patients with a poor or intermediate survival prognosis but focuses on patients with longer estimated survival times. Such a randomized trial is desirable, because the current study with strict matching criteria is retrospective in nature and may, therefore, include selection biases. However, since particularly patients with a favorable survival prognosis may be suitable candidates for upfront decompressive surgery or high-precision RT techniques, the recruitment of such a randomized trial can be exceedingly difficult [3]. Therefore, we expect that just such a trial will be very unlikely to be performed in the near future.

Patients with a slower development of motor deficits prior to RT (>14 days) did respond significantly better to RT than those patients, in whom motor deficits developed more rapidly. This can be explained by the fact that a slower development of (symptomatic) spinal cord compression results in venous congestion, which is quite likely to be reversible after treatment with RT. In contrast, a rapid progression of spinal cord compression can lead to disruption of the arterial blood flow, which may result in non-reversible infarction of the spinal cord [19].

## Conclusions

The results of 5x4 Gy and 10x3 Gy were not significantly different in in patients with MSCC and favorable survival prognoses with respect to post-RT motor function, LC of MSCC and OS. The results of the present matched-pair study would justify a randomized trial that compares 5x4 Gy and 10x3 Gy for MSCC and focuses on patients with a longer estimated survival time to develop a higher level of clinical evidence that the shorter program is optimal.

## Abbreviations

ECOG: Eastern Cooperative Oncology Group
ESTRO: European Society for Radiotherapy and Oncology
Gy: Gray
LC: Local control
MSCC: Metastatic spinal cord compression
OS: Overall survival
RT: Radiotherapy
